# Automated Chicago Classification for Esophageal Motility Disorder Diagnosis Using Machine Learning

**DOI:** 10.3390/s22145227

**Published:** 2022-07-13

**Authors:** Teodora Surdea-Blaga, Gheorghe Sebestyen, Zoltan Czako, Anca Hangan, Dan Lucian Dumitrascu, Abdulrahman Ismaiel, Liliana David, Imre Zsigmond, Giuseppe Chiarioni, Edoardo Savarino, Daniel Corneliu Leucuta, Stefan Lucian Popa

**Affiliations:** 1 Second Medical Department, “Iuliu Hatieganu” University of Medicine and Pharmacy, 400006 Cluj-Napoca, Romania; dora_blaga@yahoo.com (T.S.-B.); ddumitrascu@umfcluj.ro (D.L.D.); abdulrahman.ismaiel@yahoo.com (A.I.); lilidavid2007@yahoo.com (L.D.); popa.stefan@umfcluj.ro (S.L.P.); 2 Computer Science Department, Technical University of Cluj-Napoca, 400114 Cluj-Napoca, Romania; zoltan.czako@cs.utcluj.ro (Z.C.); anca.hangan@cs.utcluj.ro (A.H.); 3 Faculty of Mathematics and Computer Science, Babes-Bolyai University, 400347 Cluj-Napoca, Romania; imre.zsigmond@ubbcluj.ro; 4 Division of Gastroenterology, AOUI Verona, University of Verona, 37134 Verona, Italy; chiarioni@alice.it; 5 Gastroenterology Unit, Department of Surgery, Oncology and Gastroenterology, University of Padua, 35122 Padova, Italy; edoardo.savarino@unipd.it; 6 Department of Medical Informatics and Biostatistics, “Iuliu Hatieganu” University of Medicine and Pharmacy, 400349 Cluj-Napoca, Romania; dleucuta@umfcluj.ro

**Keywords:** artificial intelligence, Convolutional Neural Network, Chicago classification, Esophageal Motility Disorder Diagnosis, high-resolution esophageal manometry, machine learning

## Abstract

The goal of this paper is to provide a Machine Learning-based solution that can be utilized to automate the Chicago Classification algorithm, the state-of-the-art scheme for esophageal motility disease identification. First, the photos were preprocessed by locating the area of interest—the precise instant of swallowing. After resizing and rescaling the photos, they were utilized as input for the Deep Learning models. The InceptionV3 Deep Learning model was used to identify the precise class of the IRP. We used the DenseNet201 CNN architecture to classify the images into 5 different classes of swallowing disorders. Finally, we combined the results of the two trained ML models to automate the Chicago Classification algorithm. With this solution we obtained a top-1 accuracy and f1-score of 86% with no human intervention, automating the whole flow, from image preprocessing until Chicago classification and diagnosis.

## 1. Introduction

### 1.1. Background

High-resolution esophageal manometry (esophageal HRM) is a valuable and sophisticated diagnostic tool that revolutionized the functional evaluation of the esophagus and the esophago-gastric junction. The main indications for performing esophageal HRM are the evaluation of patients with non-obstructive dysphagia, non-cardiac chest pain, symptoms of gastroesophageal reflux disease, and the evaluation of the peristaltic reserve prior to anti-reflux surgery [1].

Esophageal HRM employs solid-state or water-perfused catheters with up to 36 circumferential pressure sensors, allowing for a simultaneous examination of the whole esophagus, from the upper esophageal sphincter (UES) to the lower esophageal sphincter (LES). Currently, esophageal HRM is the gold standard for diagnosing esophageal motility problems (EMDs). Several numerical measures, such as the integrated relaxation pressure (IRP) or the distal contractile integral, have been established and improved during the last ten years for the diagnosis of EMD (DCI). These parameters are used to characterize the LES during resting and swallowing, or the integrity and the strength of the esophageal peristaltic wave. In 2008, an international group of experts developed the first classification algorithm for EMDs based on esophageal HRM, namely the Chicago Classification [1].

Until now, with Chicago v3.0, the examination was performed in supine position, and at least 10 correct wet swallows were necessary for the final diagnosis. However, in clinical practice, there are often patients with poor compliance, and the analysis is limited to 7 or 8 clear swallows, and in these cases, sometimes, one cannot establish a definite diagnosis. To reduce the number of inconclusive diagnoses, a 4.0 version of the Chicago classification was recently proposed [2]. The protocol of the examination is longer, including also upright swallows and even solid food swallows, depending on whether or not a conclusion could be established with a high degree of certainty.

### 1.2. Related Works

The current state of the art in the automatization process of EMDs diagnosis is represented by Deep Neural Networks in Deep Learning models which can perfectly deal with non-textual data, in this case, esophageal manometry image recognition [3]. Unfortunately, although AI adoption is continuing its steady rise, the applications in esophageal manometry are limited. Only a few studies exist [3,4,5], and thus far, these applications are not used in a clinical setting.

In article [3] the authors used the variational auto-encoder model (VAE) as a generative approach to learning and then to act as a features extractor. They used Convolutional Neural Networks (CNNs) as encoder-decoder models and trained the model to automatically extract important features from HRM images. In the end, they used linear discriminant analysis (LDA) to map the selected features into a 2D space. This way they were able to define new representative features, so the manually predetermined features and the decision tree constructed by the Chicago Classification can be relaxed.

Authors from the article [4] used a more complex solution. They built a pipeline of multiple CNN models and combined the results to obtain the final diagnosis. In the first step of the pipeline, they used three CNN models to classify the images into three swallow level classes, 6 swallow types, and to classify the integrated relaxation pressure into two classes. The second step of the pipeline takes the results from these three CNNs as input. In this step, the authors added multiple different algorithms, such as XGBoost, ANN, and a rule-based model constructed based on the Chicago classification rules. Each of these algorithms was considered as sub-models and in the last step, they created a weight-based solution to combine these sub-results into the final HRM classification.

In article [5] we can see a different approach because the authors used Long-Short-Term-Memory (LSTM) instead of CNN models to classify the swallow types automatically. The accuracy of 83% obtained with this model is lower than in the case of solutions using CNNs, which can be explained by the nature of the input dataset and the LSTM, which works well mostly with time series.

Furthermore, we found two studies [6,7] that present a solution for automatic analysis of swallowing parameters of the pharynx and UES, without an automatic diagnosis for EMDs.

We mention that in our previously published study we developed an automatic classifier to assess whether the IRP is in the normal range or greater than the cut-off, and to detect the probe placement failure, based simply on the raw pictures [8]. The previous study was the first step in automating the Chicago classification process based on Machine Learning that follows the same steps as a human expert. The photos were initially pre-processed by locating the region of interest—the precise moment of swallowing [8]. Further, the photos were scaled and resized such that deep learning models could utilize them as input. To categorize the photos as successful or unsuccessful catheter positioning and to establish the precise class of the IRP, we employed the InceptionV3 deep learning model [8]. For both challenges, the trained CNN’accuracy exceeded 90%.

### 1.3. Research Gap

The Chicago scheme incorporates all the HRM parameters and is currently used worldwide for the diagnosis of EMDs. Based on the Chicago classification v3.0 [9] there are 3 main classes of EMDs: 1. Disorders with esophago-gastric junction (EGJ) outflow obstruction; 2. major disorders of peristalsis and 3. minor disorders of peristalsis. The first two classes have never been seen in asymptomatic controls. The disorders with EGJ outflow obstruction are identified based on a bnl high IRP value. In these disorders (i.e., achalasia and EGJ outflow obstruction), the LES fails to properly relax during swallows, and patients might report dysphagia, chest pain, or regurgitation. Secondary to the obstruction of the EGJ, there are also changes in the peristaltic waves. Therefore, the first diagnostic step is to rule out a disorder with EGJ outflow obstruction. Afterward, based on the aspect of the peristaltic waves (with or without breaks, spastic or not) and based on the vigor of each contraction (determined by the DCI), the major disorders of the esophageal peristalsis can be identified. If such pressure abnormalities cannot be identified, the manometry is considered normal [2]. The application of this algorithm however is not automated and requires manual analysis from the operator to correctly identify the motility pattern. This may lead to different degrees of variability in the interpretation of the tracings and eventually to the wrong diagnosis. Indeed, some studies showed that the inter-observer agreement in the diagnosis of EMDs is ‘fair-moderate’ for any diagnosis, and it is ‘substantial’ for type I and type II achalasia. In addition, the diagnostic accuracy increased with the experience of the operator [9,10]. Repeating esophageal HRM in the same patients, yielded fair reproducible results, slightly worse for peristalsis parameters [9,10,11].

Similarly, the use of artificial intelligence in esophageal HRM, could decrease the diagnostic discordance in the diagnosis of EMDs. For this reason, our study aimed to develop an automated Chicago Classification for EMDs diagnosis system using Machine Learning.

### 1.4. Contribution

In this research, we provide a technique for automating the Chicago classification process based on Machine Learning. Initially, we developed a classifier [8] based purely on the raw pressure topography photos to automatically assess whether the IRP is within the usual range or is over the cut-off. In the Chicago algorithm, determining the IRP type is one of the most crucial tasks. In the second step, we created a classifier that can differentiate between five swallowing disorders. In the last step, we implemented a simplified version of the Chicago Classification algorithm using a decision tree. The input of the decision tree is the IRP and the five different swallowing disorders and the output is the EMDs diagnosis.

The rest of this work is structured as follows: Section 2 will detail the solution we used to establish the classification pipeline, Section 3 will give some experimental findings, Section 4 will describe other methods, and Section 5 will summarize our study.

## 2. Materials and Methods

### 2.1. Raw Data Analysis

All esophageal HRM data from our manometry department were evaluated (from October 2014 to February 2021). Patients with esophageal symptoms such as dysphagia, chest discomfort, heartburn, or regurgitation were referred for manometry. We had a large number of achalasia patients since our facility is a diagnostic reference center for achalasia. The inspection process, algorithm, and categorization of EMDs were based on guidelines from Chicago v3.0, which were in use at the time. A 2-min EGJ baseline recording was followed by 10 wet swallows of 5 mL each separated by more than 30 s. Manometry was carried out early in the morning, after at least six hours of fasting, in the supine posture with the thorax angled at 30 degrees. The ISOLAB (Standard Instruments GmbH, Karlsruhe, Germany) manometry system featured a solid-state catheter with 36 sensors (Unisensor^®^, Zurich, Switzerland). This kind of catheter has an usual upper limit of IRP of 28 mmHg [2]. The catheter was inserted transnasally and at least three sensors were put in the stomach. The program indicated the moist swallows, commonly known as test swallows, with a white vertical line during the exam.

The datasets were generated by two human specialists from Romania’s “Iuliu Hatieganu” University of Medicine and Pharmacy Cluj-Napoca. Previous studies showed that the best inter-observer agreement when interpreting esophageal-HRM studies is for achalasia, while for other motility disorders the agreement is only ‘fair-moderate’ [3,4]. Therefore, the images were labeled by the Romanian specialists in collaboration with two specialists from Italy. We used Chicago classification version 3.0 because all the images included in our study were obtained between the years 2014 and 2021.

Using Chicago classification v3.0, we classified 192 esophageal HRM recordings, based on their diagnosis in: type I (27.1%), type II (19.8%) or type III (2.1%) achalasia, EGJ outflow obstruction (6.3%), absent contractility (6.8%), distal esophageal spasm (DES) (0.5%), hypercontractile esophagus (2.1%), ineffective esophageal motility (13%), fragmented peristalsis (2.1%), and normal finding (20.3%). Based on these recordings, we created two datasets.

**Figure 1:**
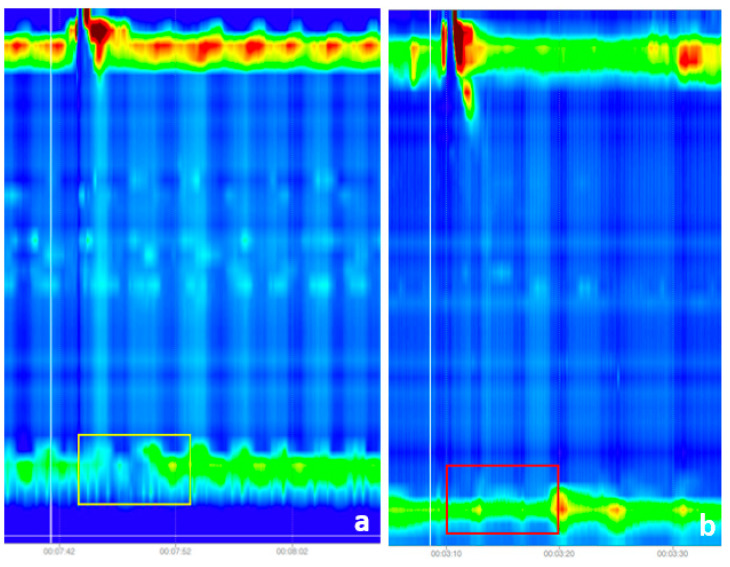
(**a**) Swallow with failed peristalsis and normal relaxation of the lower esophageal sphincter (LES), as shown by the color shift (caused by the pressure drop)—the area of focus is the yellow rectangle; (**b**) Swallow with failed peristalsis and lack of LES relaxation (there was no color change, and the measured IRP was over the cutoff—red rectangle).

The initial dataset includes photos with IRP-related labels. It comprises of 1079 photos, of which 140 had a normal IRP and 939 had an IRP greater than the threshold number. Figure 1 demonstrates instances of both normal and abnormal IRP. 

Both datasets included photos of moist swallows with simply a white vertical line (placed during the recording) indicating the test swallow. The program enables the storing of photos representing 60 s of the recording. We saved the photographs with the white mark located around the image’s center.

The IRP was measured during the first ten seconds after the commencement of the swallow, which was regarded as the white vertical line. More information about the dataset and the IRP classification algorithm can be found in our previous article [8].

The second dataset contains labeled images of six different swallowing patterns, that can be used together with the IRP to automate the Chicago classification. This dataset initially included 1535 images belonging to the following classes:

1. Panesophageal pressurization (n = 256)
2. Premature contractions (n = 27)
3. Weak contractions (n = 54)
4. Fragmented contractions (n = 58)
5. DCI (distal contractile integral) greater than 8000 mmHg·cm·s (n = 21)
6. Failed peristalsis (n = 1119)

**Figure 2:**
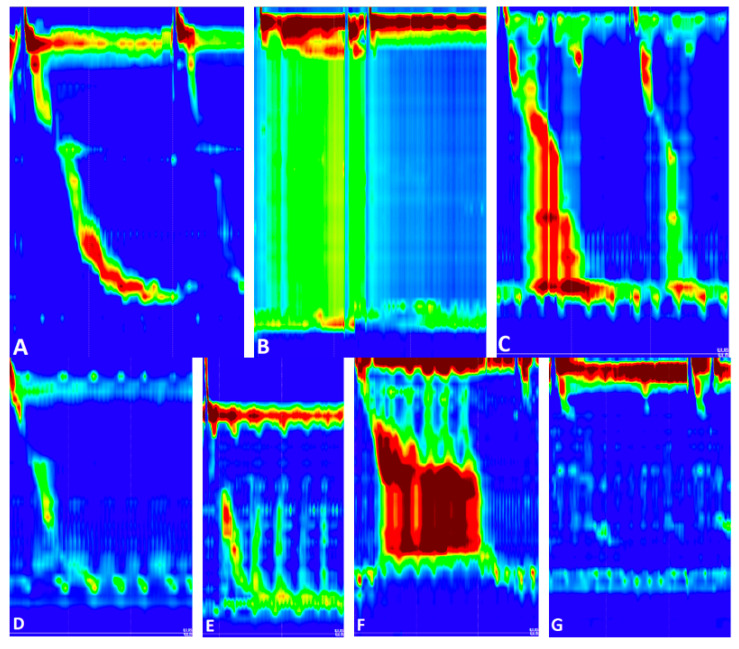
Swallowing patterns: (**A**) Normal; (**B**) Panesophageal pressurization; (**C**) Premature contraction; (**D**) Hypotensive; (**E**) Fragmented contraction; (**F**) DCI greater than 8000 mmHg·cm·s; (**G**) Failed peristalsis.

The number of failed peristalsis images was very high, due to the high number of patients with achalasia. The swallowing patterns mentioned above are presented in Figure 2. 

**Figure 3:**
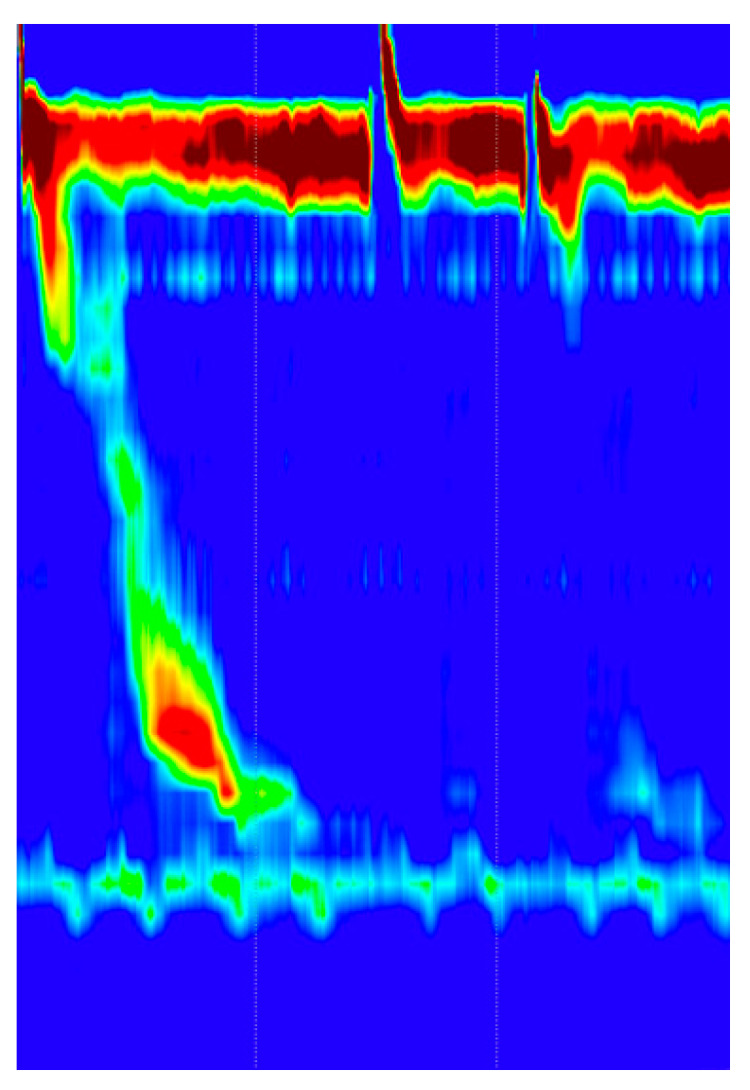
Normal swallowing pattern. The swallowing induced a strong and normal peristaltic wave.

In Figure 3 we can see an example of normal swallowing pattern.

### 2.2. Input Image Preprocessing

The raw picture contains more data than is required to train an artificial neural network, and this surplus data is referred to as noise. We removed noise from the raw images by trimming them as follows: we utilized the top, bottom, and right boundaries of the image, while the left boundary was marked by a white vertical line before each test swallow.

To pinpoint the location of the white line that runs vertically, we first created a histogram of white pixels along the y-axis and then chose the index that had the greatest pixel count. This value corresponds to the x-axis value that we wanted. The bottom section of this picture was sent to the IRP classifier as input.

Since the CNN used for IRP classification has an input shape of 299 × 299 × 3 and operates with values between −1 and 1, all pictures were rescaled and normalized to have values within the [−1, 1] range.

To classify the images in the five swallowing patterns presented above, we used the DenseNet201 CNN model [12]. This model requires 224 × 224 × 3 images this is why we rescaled the original images to this resolution and also normalized them to [−1, 1] interval. This way, from the original input dataset we obtained two different datasets, one for the IRP classification, having two classes, and one for swallowing disorder classification, having five classes.

The CNN model must be trained several times (using the training dataset) while obtaining intermediate feedback on its quality using the test dataset to build the final model. The intermediate input is used to enhance the model during the training phase. After the model has been completed, the validation dataset is used to verify the results. Having three distinct datasets guarantees that the validation set is never accessible by the model, allowing for the generation of accurate assessment scores. The training set comprises the majority of the data required to train the model. During training, the test set is used to evaluate the model’s ability to analyze images it has never seen before. During training, it is typical to continuously report metrics such as validation loss after each training phase. Since the test set is actively employed in model development and training, it is crucial to maintain a completely different collection of data. At the conclusion of the study, evaluation measures were performed on the validation set to see how well the model will perform in reality.

The final pseudocode for the Automated Chicago classification can be found in Algorithm 1.
**Algorithm 1:** Decision tree for HRM classification**If** IRP > cut-off **then**: **If** (no Panesophageal presurization images) **then**:  **Return** Achalasia Type I **Else If** (at least 2 Panesophageal presurization images):  **Return** Achalasia Type II **Else If** (at least 3 Premature contractions images):  **Return** Achalasia Type III **Else:**  **Return** EGJ Outflow Obstruction End If**Else:** **If** (at least 3 Premature contractions images) **then**:  **Return** Distal esophageal spasm **Else If** (at least 2 DCI greater than 8000 images):  **Return** Hypercontractile esophagus **Else If** (at least 5 Week images):  **Return** Ineffective esophageal motility **Else If** (at least 5 Fragmented contractions images):  **Return** Fragmented persitalsis **Else**:  **Return** Normal esophageal motility End IfEnd If

**Figure 4:**
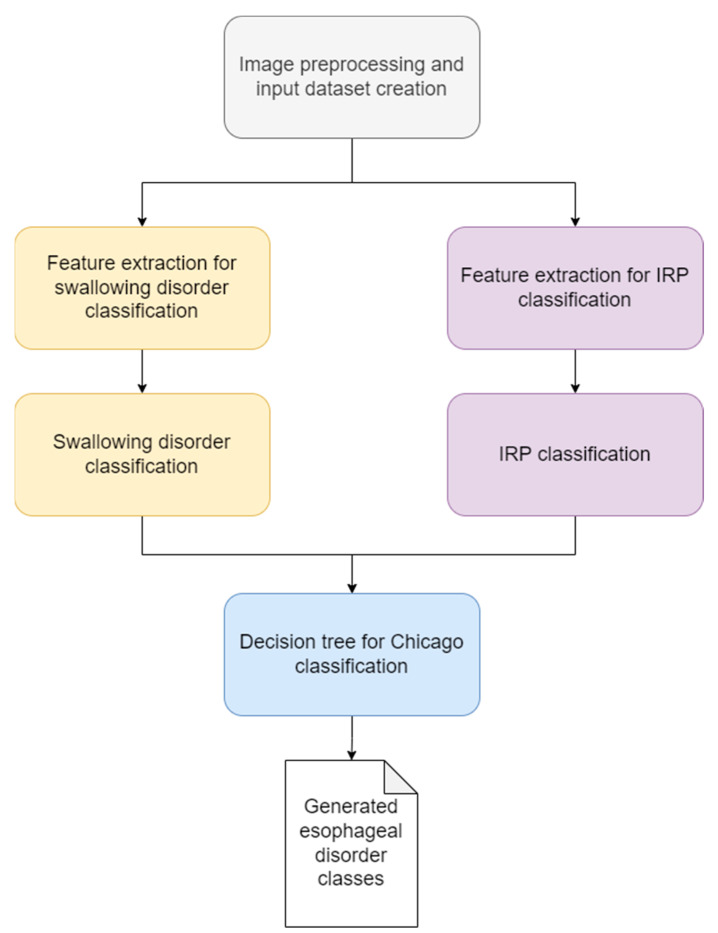
Block diagram of the final solution.

As we can see in Figure 4, the main steps of the algorithm are the following:

1. Image pre-processing and input dataset preparation: in this step, we removed the noise from the images by cropping out the unusable regions, selected the region of interest, especially for the IRP classification and we scaled and normalized the images to match the input requirements of the two CNN architectures, namely the DenseNet121 and the InveptionV3 models.
2. Feature extraction: in this step we used the DenseNet121 model for extraction of the features for the swallowing disorder classification task and the InveptionV3 model for extracting the features for the IRP classification.
3. Classification of the images: the custom fully connected layers that we added to the DenseNet121 and to the InceptionV3 models. More information can be found in the next paragraphs.
4. Decision tree for the Chicago classification: a custom-made decision tree that works on batches of images (10 images per batch) and accepts as input the class for the IRP and the class for the swallowing disorder. The output of this decision tree is the actual class of the esophageal disorder. A detailed description of this decision tree can be found in the pseudocode below.

## 3. Results

### 3.1. Solution Pipeline

Similar preprocessing procedures were used as in our prior work [11]. A simplified view of the final solution can be seen in Figure 4.

This algorithm is a continuation of our previous solution for IRP classification [8]. We extended the solution with the swallowing disorders classification part (yellow rectangles from Figure 4) and with the custom decision tree for the final step, for the classification of the esophageal disorder (blue rectangle from Figure 4).

**Figure 5:**
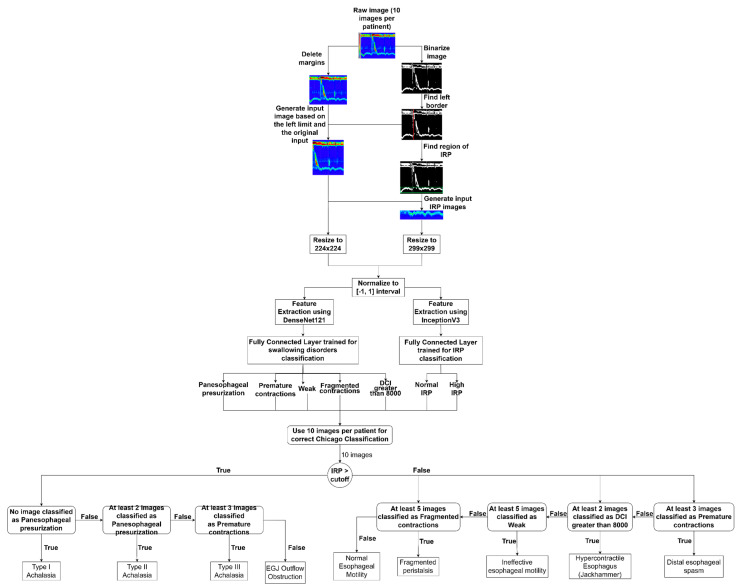
Solution pipeline.

A more detailed view can be seen in Figure 5. As shown in these figures, in the first step, we eliminated the noise by removing the top, left, and bottom margins. The image is then binarized using 128 pixels per pixel as the threshold. In this fashion, the white vertical line defining the moist swallow becomes more evident. Using the greatest value of the histogram of the white pixel described in the previous section, the x-axis location of the vertical white line is then computed.

In the subsequent picture preprocessing phase, we utilized the previously determined x coordinate to crop the original image, therefore locating the specific portion of the image that depicts a single wet swallow. This picture will be the input for the CNN classification model for swallowing difficulties. In addition, based on this picture and the binarized image, we identified the image portion that reflects the IRP for a single wet swallow. This IRP picture will serve as the input for the CNN model used to classify IRP images. After preprocessing the raw picture, we downsized the IRP images to 299 × 299, since the InceptionV3 [13] supports images of this size as input. The inputs for the swallowing disorders classifier will be adjusted to the DenseNet201 [14] CNN model’s acceptable size of 224 × 244. Next, we normalized all pixel values to the range [−1, 1], and passed the resulting matrix to the feature extraction section.

The InceptionV3 CNN model and DenseNet201 model were utilized without the final classification layer and trained on the Imagenet dataset [15,16] to extract features for the IRP classification and swallowing disorders classifier [15,16]. For IRP classification and swallowing problems classification, we developed two distinct models. To minimize overfitting issues, we chose a Global Average Layer with a 20% dropout in the final fully connected layer for the IRP classification since we only have two accessible outputs/classes and five neurons for the five swallowing disorders. A batch size of 32 pictures was used, and the data was randomized every epoch during training utilizing the Adam optimizer [17].

The results of the two trained models were used in the final part, to obtain the final diagnosis and to automate the Chicago Classification algorithm (see the Pseudocode from the previous section). The classes generated by the IRP classifier were used in the first step. If the IRP is higher than the cut-off, then we can reduce the possible esophageal disorders to 4 classes, namely Achalasia Type I, Type II, Type III, and EGJ Outflow Obstruction. In the original Chicago Classification, in the case of normal IRP, we have six different disorders, but we used only a subset of five disorders because in the case of Absent Contractility our neural network made too many mistakes, lowering the overall performance of the algorithm. In the case of normal IRP, we treated the following classes: Distal Esophageal Spasm, Ineffective Esophageal Motility, Fragmented Peristaltis, Hypercontractile Esophagus, and Normal Esophageal Motility.

In the second step/layer of the decision tree, we used the results of the second model, to classify the different swallowing disorders. To find the final result, the exact esophageal disorder, we used the following rules:

In case of IRP higher than the cut-off:

1. Achalasia Type I: if no images were classified with panesophageal pressurization
2. Achalasia Type II: if at least 2 images were classified with panesophageal pressurization
3. Achalasia Type III: if at least 3 images were classified with premature contractions
4. EGJ Outflow Obstruction: if none of the above rules were present

In case of normal IRP:

1. Distal esophageal spasm: if at least 3 images were classified as premature contractions
2. Hypercontractile esophagus: if at least 2 images were classified as DCI greater than 8000 mmHg·cm·s
3. Ineffective Esophageal Motility: if at least 5 images were classified as week
4. Fragmented peristalsis: if at least 5 images were classified as fragmented contractions
5. Normal esophageal motility: None of the above rules can be applied

A change in our algorithm compared to the Chicago classification v 3.0 is that for the Achalasia Type I we made the classification based solely on the aspect of panesophageal pressurization. We had to exclude the failed peristalsis class, because in this case, our neural network made too many mistakes, lowering the overall performance of the algorithm.

### 3.2. Metrics

To perform a thorough examination of our solution, we used many assessment criteria:

1. Accuracy: The proportion of correct classifications to the total number of instances. The automatic classification made by the neural network was compared with the diagnosis of human experts.
2. Precision: The percentage of correctly recognized positives relative to the total number of positive classifications.
3. Recall: The fraction of positives accurately detected relative to the total number of positives in the dataset.
4. F1-Score: The median between Precision and Recall.
5. Confusion Matrix: A confusion matrix summarizes the results of categorization problem prediction. The number of accurate and incorrect predictions is summed using count values and then split by class.

To appropriately compute these metrics, it is necessary to note that in the IRP classification issue, the positive class is the normal IRP class, but in the swallowing disorders classification problem, the positive class in each instance was the current disease class.

### 3.3. Integrated Relaxation Pressure Classification Results

After preprocessing the whole picture dataset and locating the IRP area of interest in each image, we trained our CNN model to categorize images as normal or high IRP. With the following assessment ratings, the outcomes of the trained Neural Network are highly encouraging:

- Accuracy—96.87%
- Precision—100.00%
- Recall—80.00%
- F1-score—88.88%

Figure 5 from our earlier work [8] depicts the confusion matrix we obtained on the test set. This matrix reveals that just one out of thirty-two photos was incorrectly identified, which is an excellent result. In our earlier research [8] we can see samples from the test set together with the anticipated label. With green, we’ve shown the correct labels, while red indicates that the CNN model misclassified the picture. Similarly, in another figure of our earlier work [8] we showed the results of the test set, with green representing accurate classification and red representing mistakes.

### 3.4. Swallowing Disorders Classification

**Figure 6:**
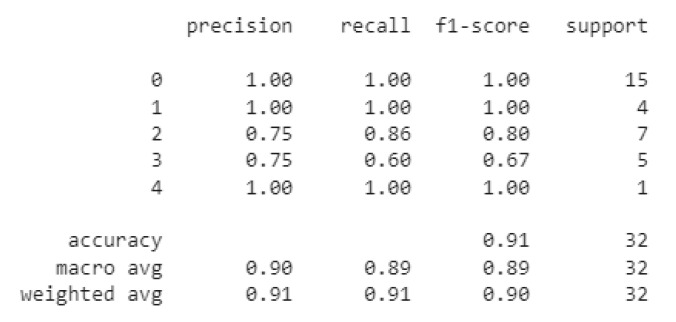

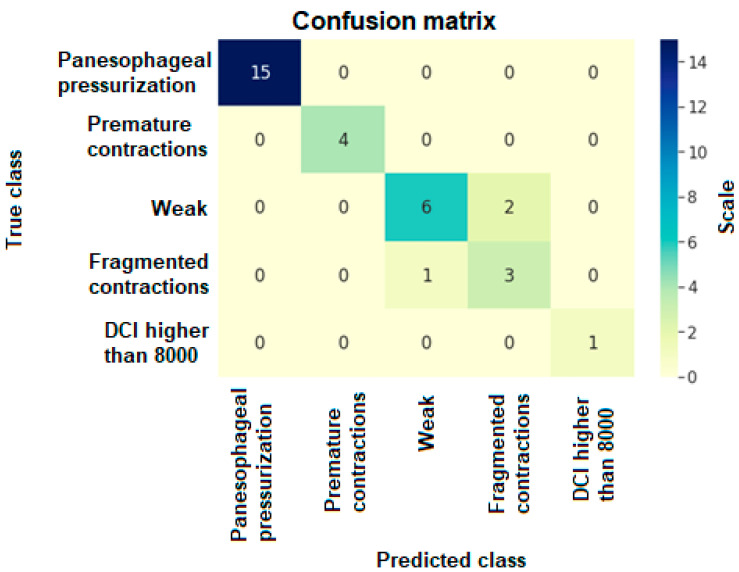
Swallowing pattern classification confusion matrix.

After running the photos through the pipeline described in the preceding section and training our CNN model, we received the confusion matrix shown in Figure 6. As shown in this matrix, the model misclassified just two of 62 photos, and we achieved the following metrics:

### 3.5. Esophageal Motility Disorders Classification

**Figure 7:**
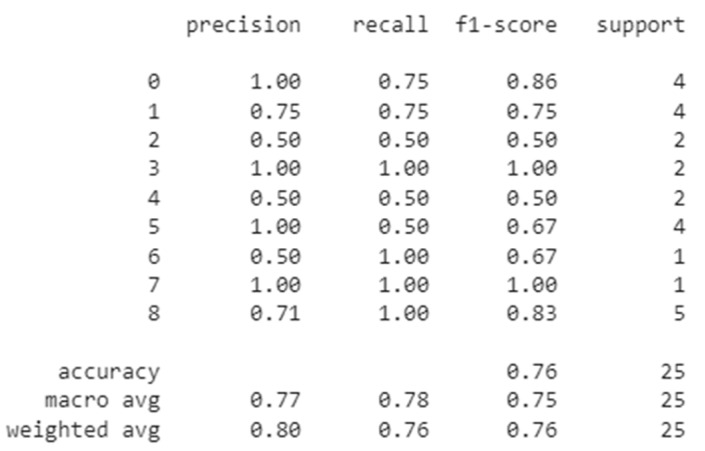

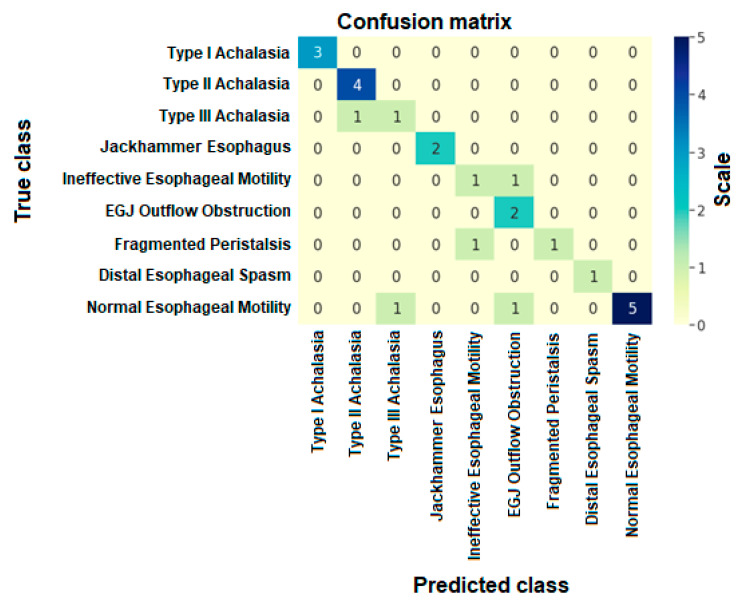
Esophageal motility disorders confusion matrix.

**Figure 8:**
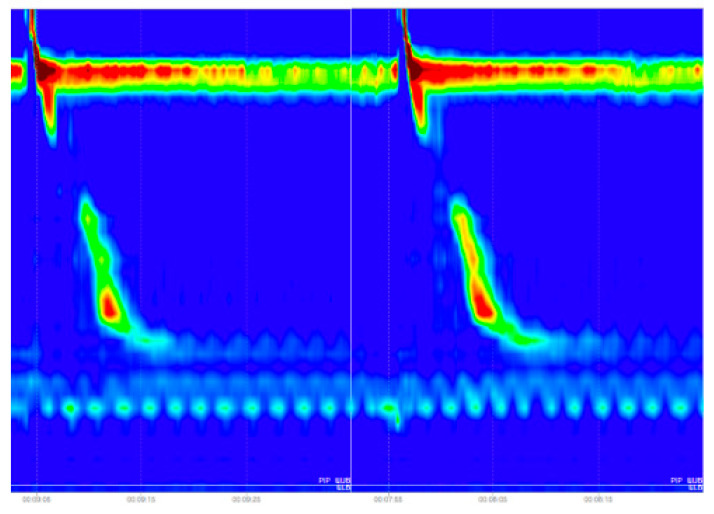
The left image depicts a week swallow, while the right image shows a fragmented contraction.

After acquiring the classification results for the IRP and the swallowing disorders the decision tree that we build for the Chicago Classification algorithm obtained the confusion matrix presented in Figure 7. The outcomes of the trained Neural Network were quite encouraging, as shown by the assessment ratings listed below:

As we can see in Figure 7, in the case of Ineffective Esophageal Motility and Fragmented Peristalsis patterns the evaluation metrics were lower than in the case of other disorders, which can be explained by the nature of these images. In case of the Fragmented Peristalsis we looked at the Fragmented Contractions swallowing pattern class and in the case of Ineffective Esophageal Motility we took into consideration the Week swallowing pattern class, but these two swallowing patterns were very similar (see Figure 8), leading the trained model to make some mistakes when trying to classify them (the inter-class similarity was very high, again explaining some of the mistakes recorded).

**Figure 9:**
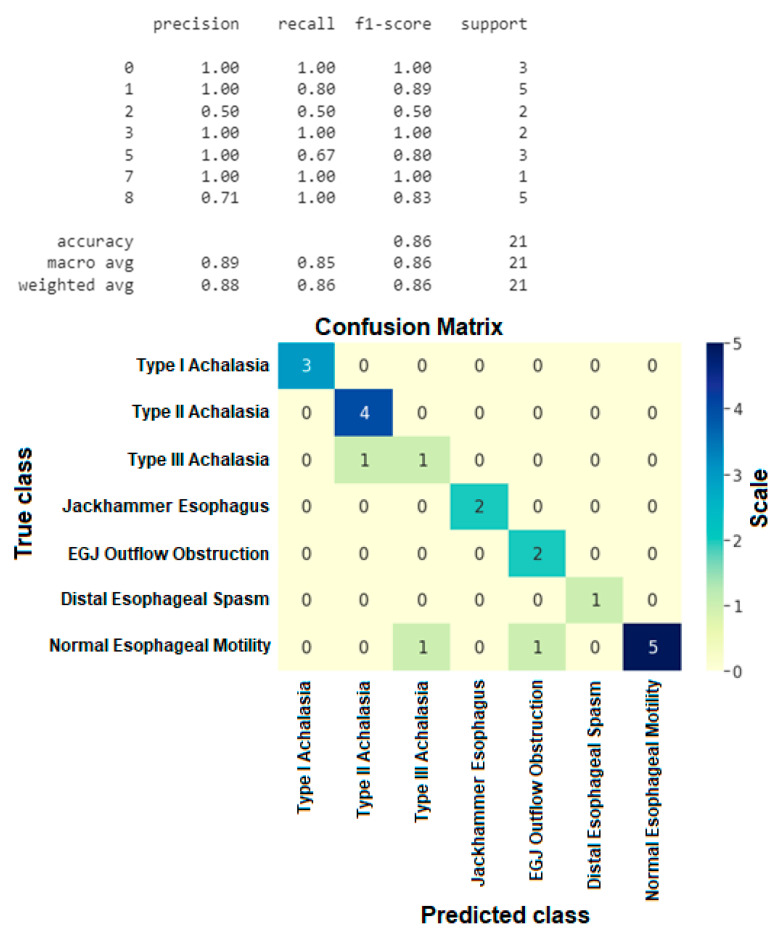
Esophageal motility disorders confusion matrix without ineffective esophageal motility and fragmented peristalsis disorder classes.

Without these two disorder classes, we had higher evaluation scores. As you can see below, we obtained an overall accuracy of 86% which, as we know, is the best top-1 accuracy so far for the fully automated Chicago classification algorithm. The final confusion matrix (with the removed swallowing classes) can be seen in Figure 9.

## 4. Discussion

In our study, a machine learning algorithm for automated diagnosis of EMDs using data extracted from esophageal HRM images is presented. A strong correlation between automatic diagnosis and the human expert diagnosis was observed, demonstrating the accuracy of the algorithm. We were able to automate the Chicago Classification method using our approach. This indicates that the final solution can categorize EMDs based on the raw photos without any input from the observer. The IRP is the most essential parameter in the Chicago Classification, thus our first goal was to assess, based purely on the raw photos, whether the IRP was within the normal range or above the cut-off. The second job was to categorize the photos into five distinct categories to differentiate between various swallowing patterns and regular patterns. In addition, we suggested a streamlined version of the Chicago Classification algorithm version 3.0 employing a decision tree structure.

The final results were affected by the inter-class similarity problem, which in some swallowing disorder classes was very high, which resulted in a lower evaluation score for some of the esophageal motility disorder classes. In the case of Ineffective Esophageal Motility and Fragmented Peristalsis patterns, the evaluation metrics were lower than in the case of other disorders, which can be explained by the nature of these images. In case of the Fragmented Peristalsis we looked at the Fragmented Contractions swallowing pattern class and in the case of Ineffective Esophageal Motility we took into consideration the Week swallowing pattern class, but these two swallowing patterns were very similar, leading the trained model to make some mistakes when trying to classify them (the inter-class similarity was very high, again explaining some of the mistakes recorded).

In the future, we will try to solve the inter-class similarities issue as we can see in the study of Li et al. [18], by adding a problem-specific feature optimization step between the feature extraction and classification layer.

There is limited research [3,4,5,19,20,21,22,23,24] that explored automated diagnosis of EMDs and pharingeal swallows utilizing AI-based methods or automation of the Chicago Classification system, Table 1. In addition, the most relevant research in this sector is discussed below.

Beginning with swallow-level raw data, Kou et al. [3] proposed an unsupervised deep learning method for automatically discovering unique esophageal motility diagnostic features and properties. In addition, the scientists constructed and trained a variational auto-encoder to categorize images into six swallow types (normal, weak, failed, fragmented, premature, and hypercontraction) and three pressurization types (normal, compartmental, panesophageal pressurization). The researchers employed a database of over 30,000 raw images of swallows, a linear discriminant approach, and then principal-component analysis to reduce the dimensionality of the data and identify the most important traits, which they then used to classify the images [3].

Another study performed by Kou et al. [4,5] on automated detection of EMDs using raw multi-swallow pictures collected from esophageal HRM, showed good accuracy by using machine learning techniques and deep-learning models with a dataset of 1741 patients.

Jell et al. [19] developed an AI-based system to test the feasibility of autonomous processing of ambulatory long-term esophageal HRM utilizing pictures from more than 900 swallows that arise during a 24-h HRM. Forty patients with suspected EMDs were recruited for the training and testing of a supervised machine learning system for automated swallow identification and categorization. The evaluation time for the whole tape was reduced from three days to eleven minutes for automated swallow detection and clustering.

In article [20] the authors extracted the pressure values measured by each transducer of the probe and they manually defined mathematical functions to interpret different physiological and mechanical phenomena. Based on these functions they built a rule-based model to classify the HRM images. With this approach, they obtained 86% accuracy, which is lower compared to our solution or other DL-based solutions.

In [21] we can read about a slightly different problem and solution. The authors of this article built a solution for real-time esophageal motility function tracing. They combined a three-dimensional CNN (Conv3D) with a bidirectional convolutional long-short-term-memory (BiConvLSTM) this way making the predictions in real-time.

It is essential to keep in mind that only a small number of studies could be compared to our inquiry, since in some studies just pharyngeal alterations and swallowing patterns were analyzed, without a comprehensive automated diagnosis of EMDs [22,23,24].

Several classification algorithms, including artificial neural networks (ANNs), multilayer perceptron (MLP), learning vector quantization (LVQ), and support vector machines, were evaluated to detect improper swallowing of the upper esophageal sphincter by Mielens et al. [22]. (SVM). The research revealed that MLP, SVM, and LVQ all exhibited high average classification accuracies, with MLP scoring 96.44%, SVM scoring 91.03%, and LVQ scoring 85.39% [22].

Talking about the limitations of our algorithm, in a real-life scenario, sometimes our algorithm confuses the Achalasia Type I class with the EGJ Outflow Obstruction class because we only used the panesophageal pressurization swallowing pattern to discriminate between these two classes. We did not use the failed peristalsis swallow pattern, because in this case, the example images had a high overlapping degree with other classes, which lowered the overall performance of the final algorithm. Therefore, some patients with ineffective esophageal motility and with absent contractility would be classified as normal. In addition, this issue will be addressed in the future by using more images, because currently, the number of images per class is imbalanced, which can introduce bias in the training of the CNN model.

We consider that our study represents a novelty compared to previous knowledge: this is the first study for automatic recognition of esophageal manometry images, which follows the same steps as a human expert.

## 5. Conclusions

This article presents a completely automated solution for the Chicago Classification method that may be used to automate the diagnosis of EMDs. In the first section, we described the preprocessing processes required to prepare input datasets. Then, we demonstrated and detailed the two distinct CNN models that we developed to categorize the IRP as normal or high and the pictures into five distinct swallowing classes. These two models were inputs for the decision tree we constructed for the Chicago classification algorithm. Achieving accuracy and an f1-score of 86% for the diagnosis of EMDs, the final findings were excellent. By automating the diagnosis of EMDs, this study may assist doctors and motility labs in their everyday work, minimize the variability between observers and save money and time on repeated duties.

## Figures and Tables

**Table 1:** Comparative results of previous studies.

Author (Year)	Number of Patients	Characteristics	Main Purpose	Outcomes	Technology
Kou et al. [3]; (2021)	2161	A generative model using the approach of variational auto-encoder was developed, for an automatic diagnosis of raw esophageal manometry data The purpose was to model and understand swallow-level data, that would be further used to develop study-level models for automatic diagnosis	To identify the swallowing type There were 6 swallow types: normal, weak, failed, fragmented, premature, or hypercontractile, and 3 pressurization types: normal, compartmental pressurization, panesophageal pressurization	The overall accuracy for the train/validation/test dataset was 0.64/ 0.63/0.64 for predicting the 6-class swallow type Overall accuracy for train/ validation/test dataset was 0.87/0.86/0.87 for predicting the 3-class swallow pressurization	DL
Kou et al. [4]; (2022)	1741	Swallow-level stage: 3 models based on Convolutional Neural Networks (CNNs) were developed to predict swallow type, swallow pressurization (classifi cation model) and integrated relaxation pressure (regression model) At the study-level stage, the models were: the rule-based model (combined with probabilities), xg-boost model and arti cial neural network (ANN)	To diagnose esophageal motility disorders Model-predicted swallow-level outcomes formed the input data of study-level models, in training and validation The blended models were weighted by precision scores.	The best performance on the test dataset, in blended models, was 0.81 in top-1 prediction, and 0.92 in top-2 prediction (xgb+ann-1)	Combines DL and ML
Kou et al. [5]; (2021)	1741	An AI-based system that automatically classifies swallow types based on raw data from HREM	To automatically classify swallow types: normal, hypercontractile, weak-fragmented, failed, and premature	Swallow type accuracies from the train/validation/test datasets of 0.86/0.81/0.83	DL
Frigo et al. [20] (2018)	226	Created a physio-mechanical model of esophageal function, and a database with parameters from healthy subjects, and different motility disorders In the first step, the relationships between the identified model parameters and pathologies were found In the second step, a decision support system was developed	Patients parameters are compared with the database and the group with the highest similarity index is chosen	Correct diagnosis in 86% of cases	Rule-based model
Wang et al. [21]; (2021)	229	A DL computational model, which leverages three-dimensional convolution and bidirectional convolutional long-short-term-memory models were used for HREM automatic diagnosis	To identify whether the esophageal function was normal, or there was a minor or major motility disorder. No final diagnosis of motility disorders was performed	Overall accuracy of the proposed model was 91.32% with 90.5% sensitivity and 95.87% specificity.	DL

AI: artificial intelligence; ANN: artificial neural networks; CNNs: Convolutional Neural Networks; DL: deep learning; ML: machine learning; HREM: high-resolution esophageal manometry.

## Data Availability

Data available on request due to restrictions eg privacy or ethical. The data are not publicly available due to the sensitive nature of medical data.
